# Association Between Endophthalmitis and the Incidence of Acute Coronary Syndrome in Patients With Ankylosing Spondylitis: A Nationwide, Population-Based Cohort Study

**DOI:** 10.3389/fimmu.2022.843796

**Published:** 2022-03-25

**Authors:** Ting-Yi Lin, Yi-Fen Lai, Wu-Chien Chien, Yi-Hao Chen, Chien-An Sun, Chi-Hsiang Chung, Jiann-Torng Chen, Ching-Long Chen

**Affiliations:** ^1^Department of Ophthalmology, Tri-Service General Hospital, National Defense Medical Center, Taipei City, Taiwan; ^2^School of Public Health, National Defense Medical Center, Taipei City, Taiwan; ^3^Department of Medical Research, Tri-Service General Hospital, National Defense Medical Center, Taipei City, Taiwan; ^4^Taiwanese Injury Prevention and Safety Promotion Association, Taipei City, Taiwan; ^5^Graduate Institute of Life Sciences, National Defense Medical Center, Taipei City, Taiwan; ^6^Department of Public Health, College of Medicine, Fu-Jen Catholic University, New Taipei City, Taiwan; ^7^Big Data Research Center, College of Medicine, Fu-Jen Catholic University, New Taipei City, Taiwan

**Keywords:** ankylosing spondylitis, acute coronary syndrome, endophthalmitis, inflammation, epidemiology

## Abstract

**Purpose:**

Ankylosing spondylitis (AS) is a risk factor for acute coronary syndrome (ACS). However, the influence of infectious insults, such as endophthalmitis, on the risk of ACS among AS patients has not been studied yet. In this study, we aimed to investigate the relationship between endophthalmitis in patients with AS and the incidence of ACS.

**Methods:**

This retrospective cohort study extracted medical records from the Taiwan Longitudinal Health Insurance Database (LHID) from January 1, 2000, to December 31, 2015. The primary outcome was the incidence of ACS. Univariate and multivariate Cox regression analyses with and without Fine and Gray’s competing risk model and Kaplan–Meier survival curve were used for the analyses. Spearman’s rank correlation coefficient was performed for sensitivity analysis.

**Results:**

We identified 530 AS patients with endophthalmitis and 2,120 AS patients without endophthalmitis for comparison. The incidence rate of endophthalmitis in our study population was 2.66%. The overall incidence rate of ACS was 1,595.96 per 100,000 person-years in AS patients with endophthalmitis and 953.96 per 100,000 person-years in AS patients without endophthalmitis (adjusted HR = 1.787; 95% CI: 1.594–2.104, *p* < 0.001). In comparison to those without comorbidities, higher adjusted HRs were found in AS patients with endophthalmitis and comorbidities such as diabetes mellitus, hyperlipidemia, hypertension, cerebrovascular accident, congestive heart failure, chronic obstructive pulmonary disease, asthma, and coronary artery disease. Besides, the age ≥ 60 years revealed a high risk for ACS in AS patients with endophthalmitis.

**Conclusion:**

Endophthalmitis was found to be an independent risk factor for ACS in patients with AS. Further clinical studies are required to elucidate the underlying mechanisms and status of systemic inflammation during endophthalmitis.

## Introduction

Ankylosing spondylitis (AS), a chronic inflammatory disorder, is a subset of spondyloarthritis. AS patients typically experience symptoms such as inflammatory back pain, morning stiffness lasting for more than 30 minutes, dull and insidious back, and buttock pain, as well as the improvement in pain with exercise and its return when inactive (1). Human leukocyte antigen (HLA)-B27 was found to be strongly associated with AS, contributing to approximately 20% of AS heritability (2). The most common extra-articular manifestations of AS are uveitis, bowel disease, lung, heart, skin, bone, and kidney involvement (3). Previous studies have reported that AS patients have an increased risk of acute coronary syndrome (ACS) (4–6).

Clinically, ACS is one of cardiovascular diseases and includes unstable angina (UA) and acute myocardial infarction (AMI). The incidence rates (IRs) of ACS were 160 to 417 per 100,000 person-years (7–10), whereas the IRs of AMI were 100 to 251 per 100,000 person-years in different countries (7, 8, 10–12). In Taiwan, the age- and gender-adjusted IR of AMI was 50.7 per 100,000 person-years in 2015 (13). ACS is potentially life-threatening and causes major healthcare and economic burden (14). The complicated pathophysiology of ACS originated rupture or erosion of coronary atherosclerotic plaques and then formed acute thrombus with intracoronary stenosis or obstruction. In addition, several studies have reported that inflammation also plays an important role in the pathogenesis of ACS (15, 16).

Endophthalmitis, an intraocular inflammatory disorder, is a severe panuveitis that can lead to persistent vision impairment or visual loss. Previous studies have reported that HLA B27-associated panuveitis could mimic endophthalmitis (17, 18). Additionally, several case reports have shown that AS patients develop endophthalmitis as a complication of systemic infection (19, 20). Furthermore, previous case reports also demonstrated the relation between endophthalmitis and cardiovascular diseases, such as myocarditis and endocarditis (21–23). However, data regarding the prevalence of endophthalmitis in AS patients are scarce. In addition, it is unclear whether endophthalmitis could influence ACS risk among patients with AS. Therefore, the epidemiological study focuses on the endophthalmitis and ACS in AS patients is an important issue. The aim of this study was to investigate the association between endophthalmitis and the incidence of ACS in patients with AS using a million-level database in Taiwan.

## Materials and Methods

### Data Source

This retrospective population-based cohort study used reimbursement data from the Longitudinal Health Insurance Database (LHID), which randomly sampled 1 million beneficiaries from the original National Health Insurance Research Database (NHIRD) in Taiwan. The representativeness of LHIDs has been validated by the National Health Research Institutes (24). More than 99% of Taiwan’s population (including foreigners) were enrolled in the single-payer National Health Insurance program. The use of NHIRD is limited to research purposes only, and researchers must follow the related laws and regulations in Taiwan. In this study, we extracted fully anonymized medical records from the LHID of 1,914,201 registered beneficiaries from January 1, 2000, to December 31, 2015. All patient demographics (including gender, age, related comorbidities, and index date) were recorded for further analyses.

### Ethical Considerations

This study was conducted in accordance with the tenets of the Declaration of Helsinki, and was approved by the Institutional Review Board of the Tri-Service General Hospital (TSGHIRB No.: B-110-56). The need for written informed consent from the study participants was waived owing to the fully anonymized data of the NHIRD.

### Patient Selection

The study population was identified using the International Classification of Disease, Ninth Revision, Clinical Modification (ICD-9-CM) codes from the LHID from 2000 to 2015. Patients who had received diagnosis of AS (ICD-9-CM code 720.0) once during hospitalization or at least three times during their outpatient visits were included. The exclusion criteria of this study were age less than 20 years; AS diagnosed before January 1, 2000; ACS before tracking; patients without tracking; or patients with incomplete medical records, defined as those having incomplete insurance status or unrelated or incorrect given codes.

Among the identified AS patients, those who had medical codes of endophthalmitis (ICD-9-CM codes 360.0, 360.00-360.04, 360.1) were further extracted as the study cohort. A comparison cohort that was four-fold the number of the study cohort and matched by gender, age, comorbidities, and index date was selected from the remaining AS patients without endophthalmitis. All patients were followed from the index date until the incidence of ACS (ICD-9-CM code of AMI: 410, and ICD-9-CM code of unstable angina: 411.1, 411.8), the date of withdrawal from the insurance system, or the end of the study period (December 31, 2015).

### Comorbidities

Patient comorbidities, including diabetes mellitus (DM), hyperlipidemia, hypertension (HTN), cerebrovascular accident (CVA), congestive heart failure (CHF), chronic obstructive pulmonary disease (COPD), asthma, coronary artery disease (CAD), cardiomegaly, and metabolic syndrome (MetS) were identified in this cohort study. Comorbidities at baseline were recorded if the patients received medical codes within 1 year before the index date with at least three medical visits. Comorbidities at the endpoint were recorded if patients received medical codes within 1 year before the incidence of ACS with at least three medical visits. The Charlson comorbidity index revised (CCI_R) score, which has been widely used to assess the presence of chronic diseases, was also recorded. The ICD-9-CM codes and definitions used in this study for data extraction and analysis are listed in **Table S1**.

### Statistical Analyses

The characteristics of AS patients with/without endophthalmitis at baseline and at the end of this study were analyzed. Continuous variables are reported as means ± standard deviations, and categorical variables are reported as numbers and percentages. The independent student t-test was used to compare continuous variables between the two study groups. Pearson chi-square and Fisher exact tests were used to evaluate the differences in the categorical variables. The cumulative risk of ACS in AS patients with/without endophthalmitis in the study period of this cohort was illustrated using a Kaplan–Meier survival curve and compared using the log-rank test. Hazard ratios (HRs) of the association between ACS and endophthalmitis, gender, age, CCI_R score, and patient comorbidities were evaluated using the univariate and multivariate Cox regression analyses. The competing-risks data are inherent to medical research in which the occurrence of the event of interest is precluded by another event. For analyzing data with competing events, we chose the measure of a proportional hazards model for the sub-distribution of a competing risk. The Fine and Gray’s competing risk model was applied to investigate the influence of all-cause mortality as a competing risk factor (25). Furthermore, sensitivity analysis with Spearman’s rank correlation coefficient was conducted to evaluate the possible multicollinearity between variables. The analyses with group stratification based on related clinical variables were also conducted. The results were reported as crude and adjusted HRs, 95% confidence intervals, and *p*-values. Statistical significance was defined as *p* < 0.05. All statistical analyses were performed using SPSS software (version 22.0; SPSS Inc., Chicago, IL, USA).

## Results

**Figure 1:**
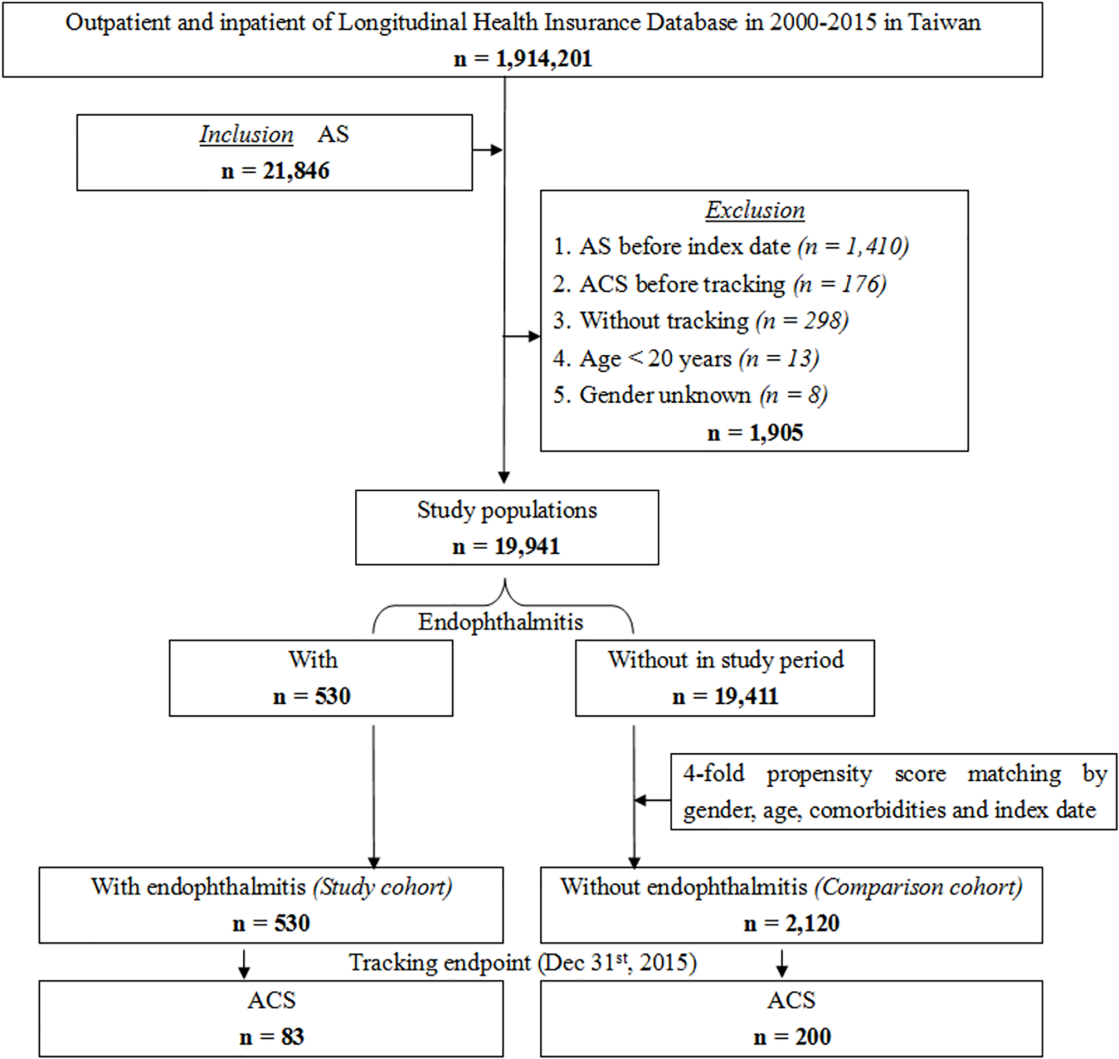
Flowchart of the patient selection in this cohort.

A total of 21,846 patients with AS were identified from the LHID of 1,914,201 registered beneficiaries between 2000 and 2015 in Taiwan. Several patients were excluded from the study for the following reasons: they were diagnosed with AS before the index date (n=1,410), ACS before tracking (n=176), without tracking (n=298), age less than 20 years (n=13), and gender unknown (n=8). Among the remaining 19,941 patients in the study population, 530 patients with endophthalmitis were selected as the study cohort. The incidence rate of endophthalmitis in our study population was 2.66%. We then retrieved a fourfold group (2,120 individuals) matched by gender, age, comorbidities, and index date from 19,411 patients without endophthalmitis as a comparison cohort (**Figure 1**).

The baseline demographic characteristics of the enrolled patients are summarized in **Table 1**. All the listed variables, namely gender, age, DM, hyperlipidemia, HTN, CVA, CHF, COPD, asthma, CAD, cardiomegaly, MetS, and CCI_R score were not significantly different between the two study groups. **Table 2** shows the demographic characteristics of the enrolled patients at the study endpoint. A total of 83 (15.66%) and 200 (9.43%) patients developed ACS in AS patients with and without endophthalmitis, respectively (*p* < 0.001). Comparing AS patients without endophthalmitis, ACS, CCI_R score, and all-cause mortality were significantly higher in AS patients with endophthalmitis. The mean follow-up period in all patients was 9.85 ± 8.52 years, and no difference was noted between the groups (**Table S2**). The mean time for ACS development after enrollment was 2.98 ± 3.27 years in the AS with endophthalmitis group, and 3.75 ± 4.16 years in AS without endophthalmitis group (*p* < 0.001; **Table S3**).

**Table 1:** The baseline demographic characteristics of the enrolled patients.

Endophthalmitis	Total		With Endophthalmitis		Without Endophthalmitis		*P*
Variables	n	%	n	%	n	%	
**Total**	2,650		530	20.00	2,120	80.00	
**Gender**							0.999
Male	1,480	55.85	296	55.85	1,184	55.85	
Female	1,170	44.15	234	44.15	936	44.15	
**Age (years)**	37.72 ± 19.80		37.69 ± 19.17		37.78 ± 19.99		0.915
**Age group (yrs)**							0.999
20-39	1,580	59.62	316	59.62	1,264	59.62	
40-59	740	27.92	148	27.92	592	27.92	
≧60	330	12.45	66	12.45	264	12.45	
**DM**							0.630
Without	1,984	74.87	396	74.72	1,588	74.91	
With	666	25.13	134	25.28	532	25.09	
**Hyperlipidemia**							0.912
Without	2,496	94.19	500	94.34	1,996	94.15	
With	154	5.81	30	5.66	124	5.85	
**HTN**							0.892
Without	2,105	79.43	420	79.25	1,685	79.48	
With	545	20.57	110	20.75	435	20.52	
**CVA**							0.946
Without	2,505	94.53	500	94.34	2,005	94.58	
With	145	5.47	30	5.66	115	5.42	
**CHF**							0.743
Without	2,577	97.25	515	97.17	2,062	97.26	
With	73	2.75	15	2.83	58	2.74	
**COPD**							0.932
Without	2,230	84.15	446	84.15	1,784	84.15	
With	420	15.85	84	15.85	336	15.85	
**Asthma**							0.890
Without	2,153	81.25	431	81.32	1,722	81.23	
With	497	18.75	99	18.68	398	18.77	
**CAD**							0.927
Without	2,486	93.81	497	93.77	1,989	93.82	
With	164	6.19	33	6.23	131	6.18	
**Cardiomegaly**							0.878
Without	2,624	99.02	525	99.06	2,099	99.01	
With	26	0.98	5	0.94	21	0.99	
**MetS**							0.946
Without	2,560	96.60	512	96.60	2,048	96.60	
With	90	3.40	18	3.40	72	3.40	
**CCI_R**	0.93 ± 1.11		0.96 ± 1.13		0.92 ± 1.11		0.175

CAD, Coronary artery disease; CCI_R, Charlson comorbidity index revised; CHF, Congestive heart failure; COPD, Chronic obstructive pulmonary disease; CVA, Cerebrovascular accident; DM, Diabetes Mellitus; HTN, Hypertension, MetS, Metabolic syndrome.

**Table 2:** Demographic characteristics of the enrolled patients at the study endpoint.

Endophthalmitis	Total		With Endophthalmitis		Without Endophthalmitis		*P*
Variables	n	%	n	%	n	%	
**Total**	2,650		530	20.00	2,120	80.00	
**ACS**							<0.001**
Without	2,367	89.32	447	84.34	1,920	90.57	
With	283	10.68	83	15.66	200	9.43	
**Gender**							0.999
Male	1,480	55.85	296	55.85	1,184	55.85	
Female	1,170	44.15	234	44.15	936	44.15	
**Age (yrs)**	41.34 ± 20.47		40.66 ± 20.23		41.50 ± 20.57		0.135
**Age group (yrs)**							0.842
20-39	1,481	55.89	295	55.66	1,186	55.94	
40-59	754	28.45	148	27.92	606	28.58	
≧60	415	15.66	87	16.42	328	15.47	
**CCI_R**	0.91 ± 1.10		0.96 ± 1.17		0.90 ± 1.10		0.011*
**All-cause mortality**							<0.001**
Without	2,417	91.21	468	88.30	1,949	91.93	
With	233	8.79	62	11.70	171	8.07	

ACS, Acute coronary syndrome, CCI_R, Charlson comorbidity index revised.

*p < 0.05. **p< 0.001.

**Figure 2:**
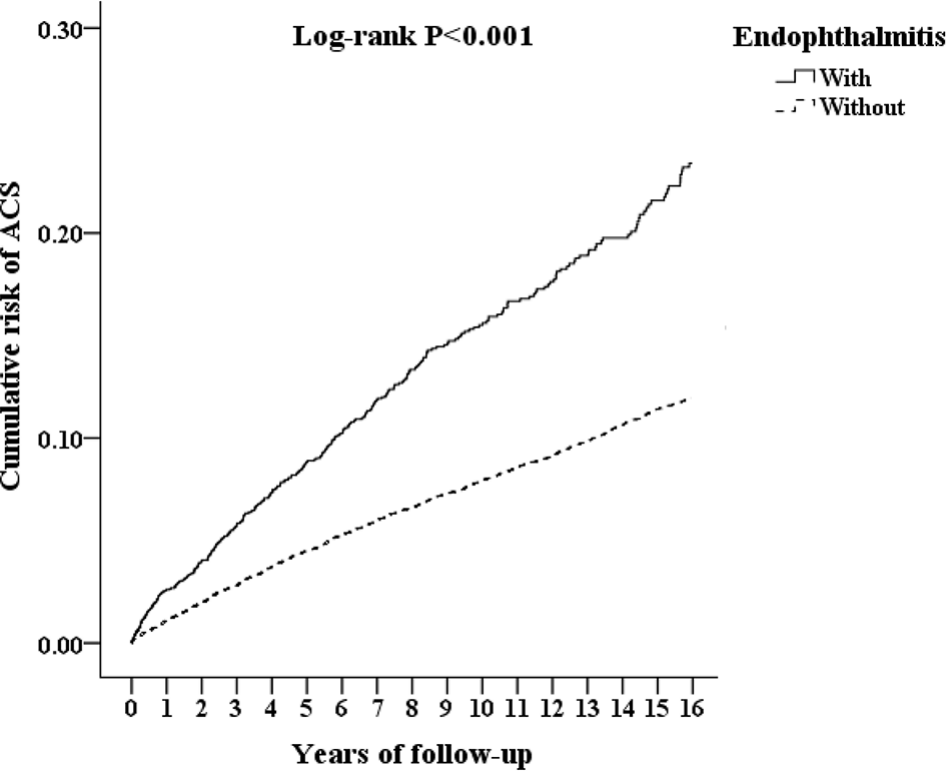
Kaplan–Meier survival curve of the cumulative risk of ACS in AS patients with and without endophthalmitis.

The Kaplan–Meier survival curve of the cumulative risk of ACS in AS patients with/without endophthalmitis is shown in **Figure 2**. A significantly higher cumulative risk of ACS was noted in AS patients with endophthalmitis than in those without endophthalmitis (*p* < 0.001; log-rank test).

**Table 3** shows the risk analysis of ACS using univariate and multivariate Cox regression with and without Fine & Gray’s competing risk model. MetS has severe multicollinearity with DM, Hyperlipidemia, or HTN (Spearman’s rank correlation coefficient r = 0.901, 0.815, and 0.832, respectively; all *p* < 0.001). This indicated that MetS has high correlation coefficients with DM, Hyperlipidemia, or HTN. Therefore, we excluded MetS from the analysis of adjusted HR in **Table 3** and **4**. After adjusting for listed variables except MetS, patients with endophthalmitis; those aged 40–59 years and ≥ 60 years, those with DM, hyperlipidemia, HTN, CVA, CHF, COPD, asthma, CAD, and those with a higher CCI_R score had a significantly higher risk of ACS (all *p* < 0.001). The statistical significance of all these risk factors persisted with or without the competing risks in the model.

**Table 3:** Risk analysis of ACS by Cox regression with/without Fine & Gray’s competing risk model.

Variables	Without competing risk model				With competing risk model			
	Crude HR (95% CI)	*P*	Adjusted HR (95% CI)	*P*	Crude HR (95% CI)	*P*	Adjusted HR (95% CI)	*P*
**Endophthalmitis**								
Without	Reference		Reference		Reference		Reference	
With	1.927 (1.617-2.147)	<0.001**	1.683 (1.497-1.935)	<0.001**	2.023 (1.733-2.188)	<0.001**	1.787 (1.594-2.104)	<0.001**
**Gender**								
Male	1.239 (0.836-1.657)	0.319	1.234 (0.819-1.638)	0.320	1.280 (0.905-1.824)	0.204	1.249 (0.867-1.810)	0.250
Female	Reference		Reference		Reference		Reference	
**Age (yrs)**								
20-39	Reference		Reference		Reference		Reference	
40-59	1.171 (1.104-1.384)	<0.001**	1.139 (1.065-1.349)	<0.001**	1.186 (1.118-1.483)	<0.001**	1.179 (1.080-1.435)	<0.001**
≧60	1.712 (1.275-1.868)	<0.001**	1.603 (1.217-1.842)	<0.001**	1.839 (1.295-1.995)	<0.001**	1.822 (1.313-1.990)	<0.001**
**DM**								
Without	Reference		Reference		Reference		Reference	
With	3.138 (2.161-5.286)	<0.001**	3.245 (2.201-5.287)	<0.001**	3.341 (2.515-5.691)	<0.001**	3.299 (2.311-5.674)	<0.001**
**Hyperlipidemia**								
Without	Reference		Reference		Reference		Reference	
With	2.872 (1.652-4.566)	<0.001**	2.814 (1.606-4.488)	<0.001**	3.127 (2.030-5.284)	<0.001**	3.145 (1.967-5.218)	<0.001**
**HTN**								
Without	Reference		Reference		Reference		Reference	
With	3.341 (2.191-5.399)	<0.001**	3.293 (2.188-5.396)	<0.001**	3.636 (2.639-6.125)	<0.001**	3.571 (2.482-5.860)	<0.001**
**CVA**								
Without	Reference		Reference		Reference		Reference	
With	2.357 (1.447-2.896)	<0.001**	2.268 (1.319-2.807)	<0.001**	2.418 (1.489-2.994)	<0.001**	2.360 (1.452-2.900)	<0.001**
**CHF**								
Without	Reference		Reference		Reference		Reference	
With	2.487 (1.622-3.120)	<0.001**	2.472 (1.573-3.066)	<0.001**	2.589 (1.689 -3.160)	<0.001**	2.501 (1.639-3.172)	<0.001**
**COPD**								
Without	Reference		Reference		Reference		Reference	
With	1.567 (1.116-2.592)	<0.001**	1.538 (1.101-2.544)	<0.001**	1.643 (1.167-2.748)	<0.001**	1.617 (1.142-2.683)	<0.001**
**Asthma**								
Without	Reference		Reference		Reference		Reference	
With	2.044 (1.138-2.814)	<0.001**	2.045 (1.107-2.762)	<0.001**	2.126 (1.211-2.915)	<0.001**	2.059 (1.197-2.899)	<0.001**
**CAD**								
Without	Reference		Reference		Reference		Reference	
With	4.434 (2.425-7.512)	<0.001**	4.341 (2.329-7.435)	<0.001**	4.588 (2.540-7.689)	<0.001**	4.425 (2.358-7.452)	<0.001**
**Cardiomegaly**								
Without	Reference		Reference		Reference		Reference	
With	0.000	0.999	0.000	0.999	0.000	0.999	0.000	0.999
**MetS**								
Without	Reference		Multicollinearity with DM, Hyperlipidemia, and HTN		Reference		Multicollinearity with DM, Hyperlipidemia, and HTN	
With	2.179 (2.048-2.270)	<0.001**			2.293 (2.169-2.563)	<0.001**		
**CCI_R**	1.281 (1.211-1.299)	<0.001**	1.246 (1.201-1.283)	<0.001**	1.294 (1.226-1.320)	<0.001**	1.276 (1.203-1.286)	<0.001**

Adjusted HR means adjusted for variables listed in the table except MetS. Competing risk: All-cause mortality.

ACS, Acute coronary syndrome; CAD, Coronary artery disease; CCI_R, Charlson comorbidity index revised; CHF, Congestive heart failure; CI, confidence interval; COPD, Chronic obstructive pulmonary disease; CVA, Cerebrovascular accident; DM, Diabetes Mellitus; HR, hazard ratio; HTN, Hypertension; MetS, Metabolic syndrome.

**p< 0.001.

**Table 4:** Risk analysis of ACS after stratification with variables listed in the table.

Endophthalmitis	With		Without *(Reference)*		Without competing risk model				With competing risk model				*P* for interaction
Stratified	Events	Rate (per 10^5^ PYs)	Events	Rate (per 10^5^ PYs)	Adjusted HR (95% CI)			*P*	Adjusted HR (95% CI)			*P*	
**Total**	83	1,595.96	200	953.96	1.683 (1.497-1.935)			<0.001**	1.787 (1.594-2.104)			<0.001**	
**Gender**													0.861
Male	47	1,618.32	109	930.65	1.717 (1.543-2.001)			<0.001**	1.812 (1.625-2.108)			<0.001**	
Female	36	1,567.68	91	983.45	1.589 (1.428-1.852)			<0.001**	1.677 (1.507-1.952)			<0.001**	
**Age (yrs)**													<0.001**
20-39	44	1,514.57	129	1,100.94	1.373 (1.235-1.601)			<0.001**	1.453 (1.304-1.685)			<0.001**	
40-59	23	1,568.65	56	938.44	1.561 (1.404-1.818)			<0.001**	1.648 (1.486-1.917)			<0.001**	
≧60	16	1,929.36	15	457.21	4.139 (3.720-4.822)			<0.001**	4.369 (3.918-5.084)			<0.001**	
**DM**													0.072
Without	64	1,647.19	183	1,067.61	1.529 (1.376-1.785)			<0.001**	1.618 (1.452-1.879)			<0.001**	
With	19	1,444.63	17	444.54	3.052 (2.744-3.557)			<0.001**	3.225 (2.889-3.747)			<0.001**	
**Hyperlipidemia**													0.135
Without	74	1,510.10	190	945.74	1.562 (1.405-1.820)			<0.001**	1.652 (1.480-1.921)			<0.001**	
With	9	2,997.10	10	1,142.53	2.980 (2.678-3.473)			<0.001**	3.148 (2.823-3.659)			<0.001**	
**HTN**													0.097
Without	65	1,574.88	188	1,057.77	1.505 (1.349-1.751)			<0.001**	1.588 (1.427-1.846)			<0.001**	
With	18	1,677.01	12	375.93	3.606 (3.238-4.203)			<0.001**	3.809 (3.415-4.429)			<0.001**	
**CVA**													0.064
Without	77	1,567.38	195	985.69	1.583 (1.420-1.844)			<0.001**	1.682 (1.495-1.942)			<0.001**	
With	6	2,083.62	5	422.93	4.189 (3.765-4.881)			<0.001**	4.425 (3.967-5.141)			<0.001**	
**CHF**													0.732
Without	81	2,164.96	199	1,101.38	1.665 (1.495-1.938)			<0.001**	1.755 (1.576-2.041)			<0.001**	
With	2	137.06	1	34.52	1.939 (1.742-2.259)			<0.001**	2.047 (1.836-2.383)			<0.001**	
**COPD**													0.674
Without	75	1,717.69	189	1,044.03	1.634 (1.469-1.905)			<0.001**	1.730 (1.547-2.005)			<0.001**	
With	8	958.90	11	384.30	2.335 (2.097-2.720)			<0.001**	2.468 (2.211-2.868)			<0.001**	
**Asthma**													0.583
Without	73	1,733.32	184	1,040.22	1.672 (1.507-1.950)			<0.001**	1.768 (1.587-2.052)			<0.001**	
With	10	1,011.07	16	488.30	1.809 (1.626-2.110)			<0.001**	1.913 (1.711-2.219)			<0.001**	
**CAD**													0.061
Without	76	1,556.53	193	973.29	1.586 (1.417-1.839)			<0.001**	1.679 (1.497-1.935)			<0.001**	
With	7	2,201.47	7	616.39	3.939 (3.542-4.590)			<0.001**	4.160 (3.728-4.838)			<0.001**	
**Cardiomegaly**													–
Without	83	1,614.04	200	957.58	1.683 (1.497-1.935)			<0.001**	1.787 (1.594-2.104)			<0.001**	
With	0	0.00	0	0.00	–			–	–			–	

Adjusted HR means adjusted for variables listed in the table. Competing risk: All-cause mortality.

ACS, Acute coronary syndrome; AMI, Acute myocardial infarction; CAD, Coronary artery disease; CCI_R, Charlson comorbidity index revised; CHF, Congestive heart failure; CI, confidence interval; COPD, Chronic obstructive pulmonary disease; CVA, Cerebrovascular accident; DM, Diabetes Mellitus; HR, hazard ratio; HTN, Hypertension. **p< 0.001.

A subgroup analysis of ACS risk between AS patients with/without endophthalmitis stratified by the listed variables was further conducted using the Cox regression with/without Fine & Gray’s competing risk model. The results are presented in **Table 4**. The overall incidence rate of ACS was 1,595.96 per 100,000 person-years in AS patients with endophthalmitis, and 953.96 per 100,000 person-years in AS patients without endophthalmitis (adjusted HR with competing risk model = 1.787; 95% CI: 1.594–2.104, *p* < 0.001). Irrespective of the patients with or without any of the stratified variables (gender, age, DM, hyperlipidemia, HTN, CVA, CHF, COPD, asthma, CAD, and cardiomegaly) and with/without competing risk in the model, the adjusted HRs were significantly elevated in AS patients with endophthalmitis than in AS patients without endophthalmitis (all *p* < 0.001 for all stratification). With respect to comorbidities, AS patients with endophthalmitis without DM, hyperlipidemia, HTN, CVA, CHF, COPD, asthma, and CAD had the following adjusted HRs for developing ACS: 1.618, 1.652, 1.588, 1.682, 1.755, 1.730, 1.768, and 1.679, respectively (all *p* < 0.001). In comparison, AS patients with endophthalmitis combined with these comorbidities had higher adjusted HRs. The adjusted HRs of AS patients with endophthalmitis and comorbidities of DM, hyperlipidemia, HTN, CVA, CHF, COPD, asthma, and CAD were 3.225, 3.148, 3.809, 4.425, 2.047, 2.468, 1.913, and 4.160, respectively (all *p* < 0.001). Among all the listed variables, the *p* for interaction showed a statistical significance between different age groups (*p* < 0.001).

## Discussion

In this study, we found a significantly higher risk of ACS in AS patients with endophthalmitis than in those without endophthalmitis after adjusting for all possible confounding factors and performing stratification analysis. According to the Kaplan–Meier analysis, endophthalmitis significantly increased the cumulative risk of developing ACS in AS patients. Besides, being in the age group of 40–59 years and ≥ 60 years; having DM, hyperlipidemia, HTN, CVA, CHF, COPD, asthma, and CAD; and having a higher CCI_R score were significant risk factors for the development of ACS in AS patients. In addition, comorbidities, including DM, hyperlipidemia, HTN, CVA, CHF, COPD, asthma, and CAD were associated with higher adjusted HRs in AS patients with endophthalmitis. Furthermore, the age ≥ 60 years was an important risk factor of ACS in AS patients with endophthalmitis.

The incidence rate of endophthalmitis was 2.66% in AS patients in our study population, which was composed of AS patients without ACS, age ≥ 20 years, and had regular follow-up medical records. Previous studies have reported that the incidence rate of endophthalmitis ranges from 0% to 16.5%, which varies among studies due to the differences in factors such as the definition of endophthalmitis, underlying etiology (post-operation, post-trauma, or dissemination from a systemic or local infection), type of surgical procedure, status of underlying disease, and whether there was illicit IV drug use (26–32). To the best of our knowledge, this is the first study to report the incidence rate of endophthalmitis among patients with AS. However, the results of our study should be interpreted carefully when comparing other incidence studies. Further prospective cohort studies are required to thoroughly investigate the incidence of endophthalmitis among patients with AS.

In this study, we obtained the data of all -cause mortality to exclude the influence of case mortality by using the Fine & Gray’s competing risk model in the study. Interestingly, the mortality rate at the study endpoint increased in AS patient with endophthalmitis. In our previous study, we also found that the mortality rate increased in endophthalmitis comorbid with renal disease, septicemia, pneumonia, and tumors (33). Thus, a further study might be needed to investigate the influence of endophthalmitis on the mortality rates in AS patients.

Age is one of important risk factors of ACS (34). In **Table 3**, significantly higher adjusted HRs of ACS were noted in age groups of 40–59 years and ≥ 60 years comparing age group of 20–39 years among AS patients (adjusted HRs = 1.179 and 1.822, respectively). Besides, **Table 4** shows that the adjusted HRs of ACS in AS patients with endophthalmitis were 1.453, 1.648, and 4.369 in 20–39 years, 40-59 years, and ≥ 60 years age groups, respectively. Furthermore, the *p* value for interaction has statistical significance between different age groups. This result indicated that the ≥ 60 years age group has higher risk of ACS than other age groups (age 20-39 and age 40-59) in AS patients with endophthalmitis. Overall, these findings might suggest that increased age could increase the risk of ACS in AS patients with endophthalmitis.

Although the etiopathogenesis of how infectious diseases increase ACS risk is unclear, many infectious etiologies have been found to have an association with ACS, including periodontitis (35), cholangitis (36), *Helicobacter pylori* (37), scrub typhus (38), syphilis (39), human immunodeficiency virus (40), herpes zoster (41), cytomegalovirus (42), hepatitis C virus (43), and coronavirus disease 2019 (44). However, none of the previous studies have investigated the relationship between endophthalmitis and ACS and the combined effect of AS and endophthalmitis on ACS risk. According to our results, the infectious insult caused by endophthalmitis was found to be an independent risk factor for ACS in AS patients. However, this result was established based on a prerequisite that correct ICD coding for ACS. Previous studies demonstrated that the positive predictive values (PPV) was 88% for the diagnosis of AMI and 47.6% for the diagnosis of CAD by using ICD coding in Taiwan’s NHIRD (45, 46). Other supporting factors [coronary intervention, stenting, antiplatelet prescription, and ATC code (C01, C07-C10)] could increase the rate of PPV by using ICD coding (45, 46). In this study, we used ICD-9-CM codes for identification of patients with ACS. Therefore, supporting factors could be used to increase the validity of the reported findings in further study.

The underlying mechanisms of atherosclerotic plaque formation and rupture are multifaceted, and involve both innate and adaptive immune responses. Lipoprotein-driven chronic inflammation of the vascular wall plays a key role in the pathophysiology of ACS (47). Many inflammatory markers, such as interleukin-1 (IL-1), IL-6, monocyte chemoattractant protein-1 (MCP-1), high-sensitivity C-reactive protein, and tumor necrosis factor (TNF)-α were identified as a surrogate of disease severity or a promising therapeutic target of ACS (48–51). In addition, the cytokine levels were found to be elevated during endophthalmitis, including the levels of granulocyte colony stimulating factor, growth-regulated oncogene, interferon gamma (IFN-γ), IL-1α, IL-1β, IL-1 receptor antagonist, IL-6, IL-8, IFN-γ-induced protein 10, MCP-1, MCP-3, macrophage inflammatory protein 1 alpha, IL-1β, transforming growth factor alpha, and TNF-α (52).

Endophthalmitis may increase the risk of ACS in AS patients through multiple mechanisms. Both infectious and non-infectious uveitis have been found to induce elevation of cytokine levels both systemically and locally (53–55). Circulating inflammatory cytokines may contribute to atherosclerosis, plaque rupture, erosion, and thrombosis by inflammatory cell recruitment, production of reactive oxygen species and proteolytic enzymes, and incitement of vascular wall damage and further inflammation (37, 47, 56). An infectious insult may, therefore, accelerate and exacerbate the condition of the cardiovascular disease, especially among susceptible individuals, such as in patients with an underlying chronic inflammatory disease. There is a scarcity of literature regarding the analyses of the systemic response to endophthalmitis. Further clinical studies are required to investigate the mechanisms by which these underlying responses contribute to an increased risk of ACS in patients with AS.

This study had several strengths. First, this study was conducted using a validated nationwide population-based million-level database in Taiwan with a 16-year follow-up period, which provides a good statistical power due to its large sample size and long follow-up period. Second, variables such as gender, age, and comorbidities were matched between the study groups. Additionally, patient comorbidities, including DM, hyperlipidemia, HTN, CVA, CHF, COPD, asthma, CAD, and cardiomegaly were adjusted during the statistical analysis. Third, univariate and multivariate Cox regression analyses were performed and Fine and Gray’s competing risk model was used to adjust for possible confounding factors in this study.

However, this study has several limitations. The causal relationship between endophthalmitis in patients with AS and ACS development was difficult to extrapolate due to the study’s retrospective cohort design. Another limitation is that all study participants were from LHID, which is largely composed of the Taiwanese population; consequently, the influence of different countries and ethnicities was not considered in this study. In our previous study, we have used ICD coding as the basis for a diagnosis of endophthalmitis to evaluate the epidemiology and mortality-related prognostic factors in endophthalmitis (33). However, these ICD codes have not been validated in National Health Insurance Research Database (NHIRD) in Taiwan. Thus, the accuracy of ICD coding for a diagnosis of endophthalmitis is one of our limitations. In addition, the disease severity, clinical features, and underlying etiologies of both AS and endophthalmitis could not be obtained from this fully anonymized database. In present study, we considered endophthalmitis as infectious events regardless of the etiologies or subtypes. However, endophthalmitis can be divided into endogenous and exogenous subtype according to the etiologies. Therefore, one limitation is that the study did not analyze the influence of endogenous and exogenous endophthalmitis on the risk of ACS in AS patients. Moreover, the medications that were prescribed were not stratified in this study, which may have influenced the incidence of ACS and endophthalmitis (either protective or exacerbating). Finally, data from laboratory examinations and medical imaging, such as X-ray, magnetic resonance imaging, or fundus images, were lacking in the NHIRD database. All study participants were identified using the ICD-9-CM codes. Misdiagnosis or coding errors cannot be completely ruled out in the current study design.

In conclusion, endophthalmitis is associated with an increased risk of ACS in patients with AS, irrespective of the listed clinical variables. Owing to increased potential cardiovascular risk, physicians should keep an eye on AS patients with a history of endophthalmitis, especially for those with underlying comorbidities. Further clinical studies are required to elucidate the underlying mechanisms and status of systemic inflammation during endophthalmitis.

## Data Availability Statement

The original contributions presented in the study are included in the article/**Supplementary Material**. Further inquiries can be directed to the corresponding author.

## Ethics Statement

The studies involving human participants were reviewed and approved by Institutional Review Board of the Tri-Service General Hospital. Written informed consent for participation was not required for this study in accordance with the national legislation and the institutional requirements.

## Author Contributions

Conceptualization: T-YL, W-CC, and C-LC. Methodology: W-CC, Y-FL, and C-HC. Software: W-CC, C-AS, and C-HC. Literature search: T-YL, and C-LC. Data acquisition and curation: W-CC, Y-FL, and C-HC. Data analysis: T-YL, W-CC, C-AS, C-HC, and C-LC. Validation: T-YL, W-CC, C-HC, and C-LC. Investigation: T-YL, Y-HC, and J-TC. Manuscript preparation: T-YL. Manuscript editing: T-YL and C-LC.Visualization: W-CC, Y-FL, and C-HC. Supervision: W-CC, Y-HC, C-AS, C-HC, J-TC, and C-LC. Project administration: W-CC and C-LC. Funding acquisition: C-LC. All authors have read and agreed to the published version of the manuscript.

## Funding

This study was supported by the Tri-Service General Hospital Research Foundation (TSGH-D-109110, TSGH-D-110112, and VTA111-V1-1-2). The sponsor has no role in study design, data collection and analysis, decision to publish, or preparation of the manuscript.

## Conflict of Interest

The authors declare that the research was conducted in the absence of any commercial or financial relationships that could be construed as a potential conflict of interest.

## Publisher’s Note

All claims expressed in this article are solely those of the authors and do not necessarily represent those of their affiliated organizations, or those of the publisher, the editors and the reviewers. Any product that may be evaluated in this article, or claim that may be made by its manufacturer, is not guaranteed or endorsed by the publisher.

## Supplementary Material

Supplementary Table 1 The ICD-9-CM codes, and definitions used in this study for data extraction and analysis.

Supplementary Table 2 Years of follow-up.

Supplementary Table 3 Years to ACS.
