# Supplementary material for: Association Between Endophthalmitis and the Incidence of Acute Coronary Syndrome in Patients With Ankylosing Spondylitis: A Nationwide, Population-Based Cohort Study
Source: Front Immunol. 2022 Mar 25;13:843796. doi: 10.3389/fimmu.2022.843796 (PMC8990883; doi:10.3389/fimmu.2022.843796)
Supplement: Supplementary Table 1 — The ICD-9-CM codes, and definitions used in this study for data extraction and analysis. [file Table_1.docx]

Supplementary Material

# Supplementary Tables

| **Table S1.** The ICD-9-CM codes, and definitions used in this study for data extraction and analysis. | |
| --- | --- |
| **Study population:** | **ICD-9-CM / Definition** |
| Ankylosing spondylitis (AS) | 720.0; Outpatient visits≧ 3 or inpatient |
| Endophthalmitis | 360.0, 360.00-360.04, 360.1 |
| **Events:** |  |
| Acute coronary syndrome (ACS) | 410, 411.1, 411.8 |
| Acute myocardial infarction (AMI) | 410 |
| Unstable angina | 411.1, 411.8 |
| **Comorbidities:** | In the baseline: 1 year before index date and medical visits ≧ 3;  In the endpoint: 1 year before ACS and medical visits ≧ 3 |
| Diabetes mellitus (DM) | 250 |
| Hyperlipidemia | 272 |
| Hypertension (HTN) | 401-405 |
| Cerebrovascular accident (CVA) | 430-438 |
| Congestive heart failure (CHF) | 428 |
| Chronic obstructive pulmonary disease (COPD) | 490-492, 494, 496 |
| Asthma | 493 |
| Coronary artery disease (CAD) | 413-414 |
| Cardiomegaly | 429.3 |
| Metabolic syndrome (MetS) | 277.7 |
| Charlson comorbidity index revised (CCI_R) | CCI removed ACS, DM, HTN, CVA, CHF, COPD, Asthma, and CAD |

| **Table S2.** Years of follow-up. | | | | | |
| --- | --- | --- | --- | --- | --- |
| **Endophthalmitis** | **Min** | **Median** | **Max** | **Mean ± SD** | ***P*** |
| Total | 0.01 | 7.73 | 15.99 | 9.85 ± 8.52 | 0.812 |
| With | 0.01 | 7.68 | 15.99 | 9.80 ± 8.41 |  |
| Without | 0.01 | 7.77 | 15.99 | 9.88 ± 8.55 |  |
| *P*: t-test | | | | | |

| **Table S3.** Years to ACS. | | | | | |
| --- | --- | --- | --- | --- | --- |
| **Endophthalmitis** | **Min** | **Median** | **Max** | **Mean ± SD** | ***P*** |
| Total | 0.03 | 2.30 | 15.81 | 3.53 ± 4.01 | <0.001 |
| With | 0.03 | 2.01 | 15.74 | 2.98 ± 3.27 |  |
| Without | 0.03 | 2.52 | 15.81 | 3.75 ± 4.16 |  |
| *P*: t-test | | | | | |
